# First-line treatment of hepatocellular carcinoma: a propensity-matched analysis of tyrosine kinase inhibitors combined with TACE, with or without PD-1 inhibitors

**DOI:** 10.3389/fphar.2025.1533471

**Published:** 2025-05-13

**Authors:** Yanjun Shen, Yawen Xu, Ying Teng, Xiaoyan Ding, Jinglong Chen

**Affiliations:** Department of Oncology, Beijing Ditan Hospital, Capital Medical University, Beijing, China

**Keywords:** hepatocellular carcinoma, transarterial chemoembolization, tyrosine kinase inhibitor, immune checkpoint inhibitor, propensity scores

## Abstract

**Objective:**

This study attempted to comprehensively assess the clinical outcomes of cases with progressive HCC (pHCC) undergoing treatment with TKI and ICI in conjunction with TACE, as compared to the combination of TKI with TACE alone.

**Methods:**

From March 2019 to January 2022, this cohort comprised 82 cases who received TACE in conjunction with TKI and 52 cases who were treated with TACE plus TKI alone. The propensity scores was used to mitigate selection bias.

**Results:**

The multivariate analysis further reinforced that liver cirrhosis (HR = 1.233, 95% CI: 1.024–1.484, P = 0.027), tumor diameter (HR = 1.283, 95% CI: 1.086–1.515, P = 0.003), and the treatment strategy (HR = 0.495, 95% CI: 0.264–0.793, P = 0.000) were independently linked to OS, underscoring their prognostic relevance.

**Conclusion:**

Incorporating TACE, TKI, and ICI remarkably enhanced both PFS and OS relative to TACE with TKI alone, positioning it as a more efficacious first-line therapeutic strategy for unresectable HCC, while maintaining an acceptable safety profile in clinical settings.

## Introduction

Hepatocellular carcinoma (HCC) represents a highly aggressive neoplasm, with profoundly elevated rates of both morbidity and mortality (Freddie et al., 2024; Llovet et al., 2022). The predominant etiological factors underlying the development of HCC are chronic viral hepatitis and the inexorable progression of liver cirrhosis (Daniel et al., 2023 **)**. Alarmingly, more than 80% of HCC cases are diagnosed at intermediate or advanced stages, where curative options are limited (Henrik et al., 2020; Ju Dong et al., 2019). Transarterial chemoembolization (TACE) is regarded as the standard therapeutic modality for intermediate HCC and remains the most extensively utilized intervention for cases with intermediate-to-advanced disease (Jean-Luc et al., 2019; European Association for the Study of the Liver, 2018).

Notably, the advent of targeted pharmacotherapies, involving sorafenib, donafenib, and Lenvatinib (LEN)—alongside immunotherapeutic strategies, has regarded as first-line approaches for advanced HCC (Zhou et al., 2023; Benson et al., 2021 **)**. The combinatorial regimen of atezolizumab and bevacizumab has been substantiated through clinical evidence as both efficacious and well-tolerated in advanced cases (Finn et al., 2020). Moreover, the synergistic integration of immune-based therapies with TACE holds promise for further clinical benefit, as TACE potentiates the release of tumor antigens and enhances immune recognition (Masatoshi et al., 2023).

Given the remarkable tumor burden, the presence of portal vein tumor thrombus (PVTT), and the frequent occurrence of impaired hepatic function in unresectable HCC cases, monotherapeutic approaches mainly demonstrate suboptimal efficacy. In this context, it has been demonstrated that the combination of LEN and TACE significantly augmented survival outcomes in HCC cases (Jun-Ning et al., 2023), Consequently, the therapeutic regimen of TACE, a tyrosine kinase inhibitor (TKI), and an immune checkpoint inhibitor (ICI) has proven to be as a potentially advantageous strategy. Nevertheless, the clinical efficacy of this combined modality has not yet been documented.

Therefore, predicated upon the hypothesized synergistic mechanisms of action, in the present investigation, it was attempted to figure out the clinical outcomes of cases with progressive HCC (pHCC) undergoing treatment with TKI and ICI in conjunction with TACE, as compared to the combination of TKI with TACE alone.

## Materials and methods

### Patients

Clinical data were precisely gathered from a cohort of 134 HCC cases who underwent treatment at Beijing Ditan Hospital, Capital Medical University, between March 2019 and January 2022. This cohort comprised 82 cases who received TACE in conjunction with TKI and 52 cases who were treated with TACE plus TKI alone. The study protocol received ethical approval from the relevant Institutional Ethics Committee and was implemented in accordance with the principles articulated in the Declaration of Helsinki. Informed consent was duly attained from all participants for the utilization of their clinical data in this investigation.

The inclusion criteria for this investigation were summarized as follows: 1) cases must receive either TACE plus TKI or TACE plus TKI with ICI as their first-line therapeutic intervention; 2) liver function prior to treatment was required to be classified as Child-Pugh Class A or Class B; 3) cases must be at Barcelona Clinic Liver Cancer (BCLC) stage B or C; 4) a complete absence of other malignancies was mandated; 5) participants were required to be between the ages of 18 and 80 years; 6) an Eastern Cooperative Oncology Group Performance Status (ECOG PS) score ranging from 0 to 1 was necessary; and 7) the necessity of at least one target lesion with a measurable diameter of ≥1 cm.

Conversely, the exclusion criteria were delineated as follows: 1) cases with prior treatment histories for HCC, including whereas not limited to surgical intervention, ablation, or radiotherapy; 2) cases exhibiting coagulation disorders; 3) cases with a concurrent diagnosis of other malignancies; and 4) those who experienced gastric or esophageal variceal hemorrhage within the preceding month.

### Clinical parameters and laboratory results

Peripheral blood samples were attained from cases between 7:30 and 9:30 AM, precisely 1 week prior to the commencement of combination therapy. A comprehensive array of clinical, laboratory, and radiological data was systematically extracted from medical record systems. This dataset involved variables, comprising age, gender, ECOG PS score, BCLC stage, Child-Pugh classification, alpha-fetoprotein levels, tumor distribution, size, multiplicity, presence of liver cirrhosis, vascular invasion, extrahepatic metastasis, as well as various hematological and biochemical indices.

## Treatment

### TACE procedure

All cases underwent synchronous TACE, the methodology of which has been elucidated in prior investigations (Yanjun et al., 2019). In brief, the TACE procedure was implemented by adept interventional radiologists, who delivered iodine oil (5–10 mL) in conjunction with one or more chemotherapeutic agents, involving pirarubicin, hydroxycamptothecin, or lobaplatin. Subsequently, embolization was executed utilizing embolic materials, involving gelatin sponge particles or polyvinyl alcohol particles, until complete stasis in the tumor-feeding vasculature was attained.

### Administration of TKI and ICI

TKIs and ICIs were strategically withheld for a period of 5 days preceding and following the TACE intervention. The PD-1 inhibitors—specifically camrelizumab (200 mg), tislelizumab (200 mg), toripalimab (200 mg), or sintilimab (200 mg)—were administered via intravenous infusion once every 3 weeks.

In accordance with the physician’s assessment regarding drug tolerance, particularly in the context of advanced age, non-Child-Pugh A classification, diminished body weight, the presence of ascitic fluid, and gastrointestinal varices with a potential for hemorrhage, the oral TKI regimens included sorafenib (400 mg daily or 400 mg twice daily), LEN (8 mg or 4 mg daily), or apatinib (0.25 g daily), all delivered in 21-day cycles.

It was attempted to precisely evaluate adverse events (AEs), employing the Common Terminology Criteria for Adverse Events (CTCAE) version 4.03(UMLS metathesaurus - NCI_CTCAE, 2020). The modification or cessation of TKI or ICI therapies was executed under physicians’ vigilant guidance. Furthermore, all cases with active hepatitis B virus (HBV) infection were provided with oral antiviral therapy as a standard precaution.

### Follow-up and assessments

The initial diagnosis of HCC was on the basis of the criteria released by the American Association for the Study of Liver Diseases (Bruix and Sherman, 2011). Tumor evaluation was systematically undertaken every 4–6 weeks, with therapeutic responses assessed through triphasic scanning techniques, MRI, or CT, in accordance with the modified Response Evaluation Criteria in Solid Tumors (mRECIST) (Yu et al., 2022). The primary endpoints encompassed overall survival (OS) and progression-free survival (PFS). Definition of OS included the duration from the first administration of combination treatment to the date of mortality from any cause or to the last follow-up (July 31, 2022). PFS was characterized as the interval from the initial use of combination therapy to the occurrence of tumor progression or mortality. The tumor progression or unacceptable adverse reactions, the patient received second-line treatment. The second-line therapy included other targeted drug, regorafenib, or radiotherapy, or HAIC (Hepatic Artery Infusion Chemotherapy).

### Statistical analysis

Propensity scores were computed to facilitate patient matching, employing a caliper of 0.02 in a 1:1 ratio utilizing a logistic regression model. The propensity scores used the following variables: age, gender, BCLC stage, Child-Pugh classification, alpha-fetoprotein levels, tumor distribution, size, presence of liver cirrhosis, vascular invasion, extrahepatic metastasis. Data analysis was implemented through SPSS 22.0 software that was developed by IBM Corp. It was attempted to plot Kaplan–Meier survival curves via GraphPad Prism 7.0 software that was developed by GraphPad Software Inc. Comparisons particularly between the two principal groups were executed using Student’s t-test for continuous variables, while the analysis of categorical variables was undertaken through Pearson’s χ^2^ test with continuity correction. Survival rates were estimated at each time point through Kaplan–Meier curve analysis. For multivariate regression analysis, a Cox proportional hazards model was implemented. Definition of statistical significance was through P-value falling behind 0.05.

## Results

### Patients’ characteristics

In the study, a cohort of 134 HCC cases was recruited, involving 52 cases allocated to the T+T group and 82 to the T+T+I group, as detailed in Table 1. In the T+T+I cohort, all cases received TKI, comprising LEN (n = 70), sorafenib (n = 8), and apatinib (n = 4), in conjunction with a programmed cell death-1 (PD-1) inhibitor: sintilimab (n = 45), camrelizumab (n = 31), toripalimab (n = 4), and tislelizumab (n = 2). Conversely, in the T+T cohort, 28 cases were administered LEN, while the remaining 24 received sorafenib. Post-propensity score matching (PSM), 102 cases were incorporated into the balanced cohort, involving 51 cases per group, ensuring comparable baseline characteristics between the two groups (Table 1). Regarding metastatic dissemination, the T+T+I group exhibited pulmonary metastases in 9 cases, lymph node metastases in 15, bone metastases in 8, and other forms of metastasis in 17 cases. Meanwhile, the T+T group demonstrated pulmonary metastases in 11 cases, lymph node involvement in 9, bone metastases in 3, and other metastatic manifestations in 9 cases (Table 2).

**TABLE 1:** Patients’ baseline characteristics in the two principal groups pre- and post-PSM.

	Triple therapy group n = 82	Control group n = 52	P	Triple therapy group n = 51	Control group n = 51	P
Sex (M/F)	65/17	44/8	0.334	43/8	44/7	0.78
Age,< 55/≥55	29/53	18/34	0.929	16/35	17/34	0.832
Albumin (g/L)	37.81 ± 4.32	38.40 ± 4.72	0.416	37.74 ± 4.12	38.36 ± 4.73	0.485
Total bilirubin (lmol/L)	19.05 ± 8.80	17.88 ± 8.57	0.453	19.92 ± 8.78	17.99 ± 8.63	0.264
Lymphocyte	1.18 ± 0.51	1.08 ± 044	0.194	1.17 ± 0.49	1.08 ± 0.45	0.327
Neutrophil	3.39 ± 1.92	2.85 ± 1.28	0.074	3.24 ± 2.03	2.85 ± 1.29	0.248
PT(s)	15.99 ± 2.29	12.66 ± 1.59	0.413	12.95 ± 1.69	12.67 ± 1.61	0.403
PLT(X109/L)	131.02 ± 68.04	112.96 ± 58.20	0.116	135.01 ± 11.27	114.23 ± 8.13	0.138
AFP (ng/ml) <400/≥400	50/32	35/17	0.458	33/18	34/17	0.835
Child-pughclass (A/B)	63/19	37/15	0.462	38/13	36/15	0.657
Liver cirrhosis (Yes/No)	65/17	37/15	0.283	40/11	36/15	0.363
Tumor diameter(cm) ≤5/>5	31/51	24/28	0.068	18/33	24/27	0.227
No. of nodules, n (%) (≤3/> 3)	54/28	32/20	0.612	34/17	31/20	0.537
PVTT(Yes/No)	50/32	30/22	0.706	26/25	30/21	0.426
BCLC stage (B/C)	20/62	16/36	0.417	16/35	16/35	1
Extrahepatic spread (Yes/No)	30/52	25/27	0.188	19/32	25/26	0.23
TACE period	4(2.0,7.0)	4(2.0,6.5)	0.291	4(1.8,6.9)	4(2.0,6.8)	0.319

**TABLE 2:** Baseline characteristics of tumors.

	Triple therapy group	Control group
Tumor diameter (cm)		
Median value		
<3	11	4
3–5	18	20
5–10	39	25
≥10	14	3
Lobar location(L/R)	34/48	15/37
Metastasis sites		
Lung	9	11
Lymph nodes	15	9
Bone	8	3
Other	17	9
Portal vein tumor thrombus, n (%)		
Absent	32	22
Vp-1 and 2	28	16
Vp-3	17	11
Vp-4	5	3
Etiology		
Hepatitis B	72	45
Hepatitis C	8	5
Other	3	3

### Efficacy

**FIGURE 1:**
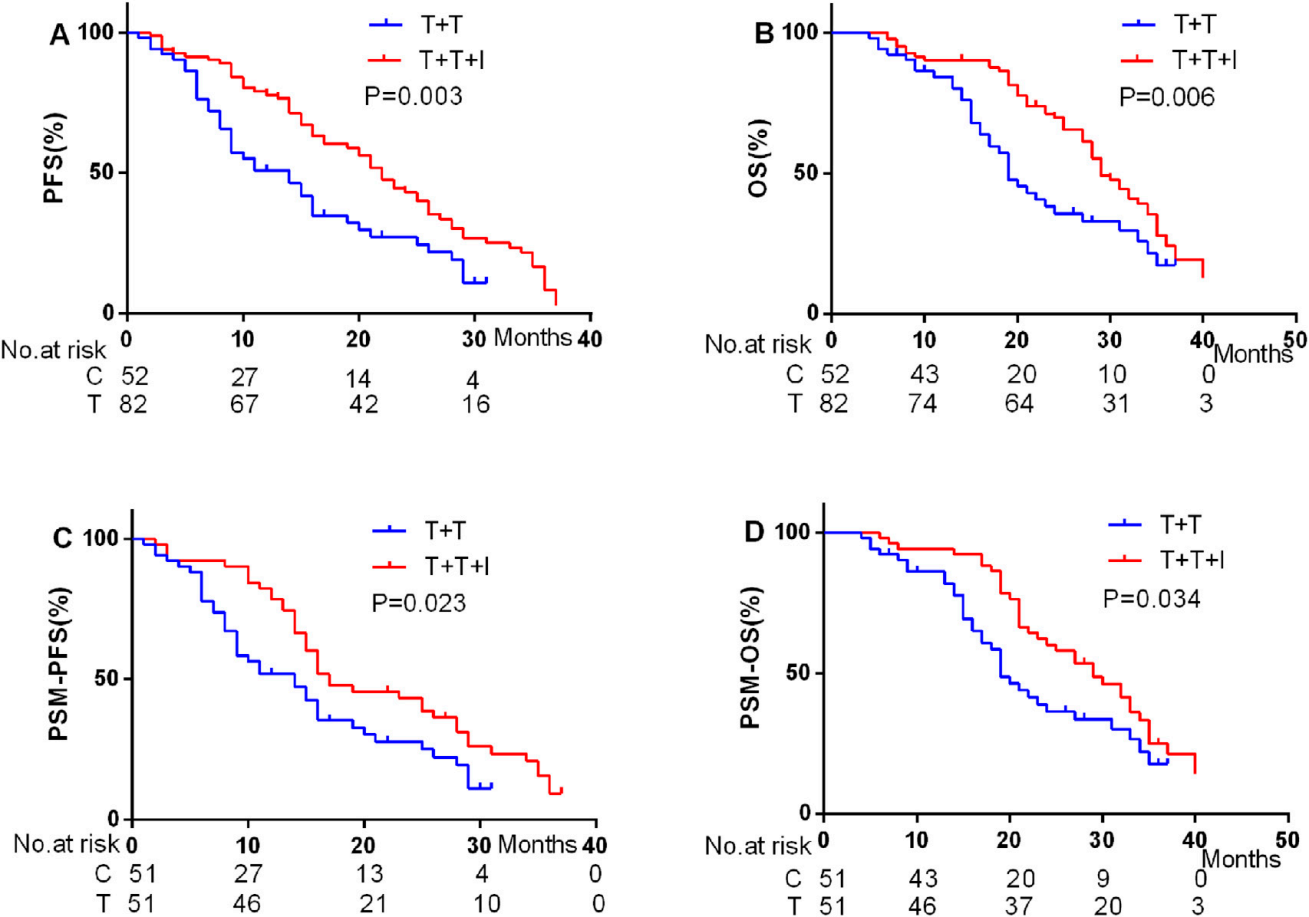
PFS and OS before **(A, B)** and after **(C, D)** PSM.

It was attempted to implement a comparative assessment of the best overall response between the two cohorts. According to the modified RECIST (mRECIST) criteria, the overall response rate (ORR) was notably higher particularly in the T+T+I group, recorded at 54.90% prior to PSM and elevated to 66.70% post-PSM. In contrast, the ORR in the T+T group was 55.70% prior to PSM and 57.80% following PSM, demonstrating a relatively smaller variation in response (Table 3). In terms of survival outcomes, prior to PSM, the median PFS (mPFS) and median OS (mOS) were 20 and 29 months, respectively, in the T+T+I group, relative to 12 and 19 months in the T+T group (P = 0.003 for mPFS; P = 0.006 for mOS) (Figures 1A, B). Following PSM, the T+T+I group continued to demonstrate superior survival outcomes, with mPFS and mOS of 17 and 28 months, respectively, relative to 12 and 19 months in the T+T group (P = 0.023; P = 0.034) (Figures 1C, D).

**TABLE 3:** Overview of outcomes on the basis of the mRECIST criteria.

	Before PSM			After PSM		
	Triple therapy group	Control group	P	Triple therapy group	Control group	p
Response of initial treatment	82	52		51	51	
CR	8	4		6	4	
PR	37	25		28	25	
SD	30	14		16	13	
PD	7	9		1	9	
ORR	54.90%	55.70%	**0.651**	66.70%	56.80%	**0.911**

### Analysis of prognostic factors

To further elucidate the independent determinants influencing OS and PFS, it was attempted to execute univariate and multivariate Cox proportional hazards regression analyses following PSM (Table 4). The multivariate analysis unveiled that BCLC stage (HR = 1.940, 95% CI: 1.560–2.413, P = 0.000) and the therapeutic regimen (HR = 0.67, 95% CI: 0.291–0.962, P = 0.000) emerged as significant independent prognostic factors for PFS.

**TABLE 4:** Cox proportional hazards univariate and multivariate analysis for PFS and OS in the matched cohort.

	Progression-free survival				Overall survival			
	Univariate analysis		Multivariate analysis		Univariate analysis		Multivariate analysis	
	HR (95% CI)	p	HR (95% CI)	p	HR (95% CI)	p	HR (95% CI)	p
Gender(M/F)	0.976(0.825–1.154)	0.084			0.965(0.842–1.107)	0.613		
Age (<55/≥55,years)	1.153(0.910–1.317)	0.305			0.968(0.865–1.083	0.572		
Albumin (≤35/>35 g/L)	0.917(0.704–1.155)	0.108			0.931(0.613–1.102)	0.131		
TBIL (≤34.2/>34.2lmol/L)	0.933(0.833–1.046)	0.232			0.937(0.825–1.177)	0.402		
Lymphocyte(≤1.1/>1.1 X109/L)	0.917(0.750–1.120)	0.396			0.914(0.791–1.056)	0.222		
Neutrophil(≤1.8/ >1.8 × 109/L)	0.899(0.701–1.211)	0.227			0.950(0.696–1.298)	0.749		
PT(≤14/>14s)	0.933(0.833–1.046)	0.232			1.071(0.898–1.277)	0.445		
PLT(≤100/> 100 × 109/L)	0.819(0.617–1.3110	0.423			0.907(0.605–1.206)	0.302		
AFP (≤400/>400 ng/ml)	1.057(0.854–1.309)	0.608			1.842(1.718–1.9980	0.035		
Child-pugh class(A/B)	0.884(0.720–1.804)	0.236			0.983(0.785–1.270)	0.365		
Liver cirrhosis (Yes/No)	0.877(0.765–1.006)	0.061			1.179(1.052–1.322	0.005	1.233(1.024–1.484)	0.027
Tumor diameter (≤5/>5cm)	1.126(0.723–1.846)	0.318			1.604(1.408–1.746)	0.000	1.283(1.086–1.515)	0.003
No. of nodules (≤3/> 3)	1.169(0.903–1.514)	0.139			1.088(0.952–1.243)	0.216		
PVTT(Yes/No)	0.881(0.703–1.104)	0.272			1.040(0.898–1.210)	0.354		
BCLC stage (B/C)	1.812(1.475–2.227)	0.000	1.940(1.560–2.413)	0.000	1.023(0.924–1.134)	0.658		
Extrahepatic spread (Yes/No)	0.829(0.729–1.543)	0.346			0.918(0.835–1.070)	0.079		
Treatment	0.469(0.163–0.856)	0.001	0.67(0.291–0.962)	0.000	0.425(0.268–0.602)	0.000	0.495(0.264–0.793)	0.000

Moreover, the multivariate analysis further reinforced that liver cirrhosis (HR = 1.233, 95% CI: 1.024–1.484, P = 0.027), tumor diameter (HR = 1.283, 95% CI: 1.086–1.515, P = 0.003), and the treatment strategy (HR = 0.495, 95% CI: 0.264–0.793, P = 0.000) were independently linked to OS, underscoring their prognostic relevance.

### Safety analysis

Before PSM, the incidence of treatment-related AEs between the two principal groups is summarized in Table 5. Importantly, no treatment-related mortalities were reported in either cohort. The most prevalent treatment-related AEs of any grade included an increase in aspartate aminotransferase (AST) (n = 68, 82.92% in the T+T+I group; n = 35, 67.31% in the T+T group), an increase in alanine aminotransferase (ALT) (n = 52, 63.41% in the T+T+I group; n = 28, 53.84% in the T+T group), pyrexia (n = 40, 48.78% in the T+T+I group; n = 34, 65.38% in the T+T group), and hypertension (n = 35, 42.68% in the T+T+I group; n = 21, 40.38% in the T+T group). Regarding grade 3 treatment-related AEs, elevated ALT level was observed in 19.51% (n = 16) of patients in the T+T+I group and 13.46% (n = 7) in the T+T group, while elevated AST level was noteworthy in 18.29% (n = 15) and 15.38% (n = 8) of patients in the respective groups. These AEs were transient in nature, generally resolving in a short timeframe following the cessation or adjustment of treatment.

**TABLE 5:** Treatment-related adverse events.

	Any grade		Grade 3–4	
	Triple Therapy Group N = 82	Control Group N = 52	Triple Therapy Group N = 82	Control Group N = 52
Hypertension	35	21	6	4
Aspartate aminotransferase increase	68	35	15	8
Alanine aminotransferase increase	52	28	16	7
Blood bilirubin increase	8	5	2	0
Fatigue	23	15	3	1
Proteinuria	16	9	4	2
Diarrhea	33	19	4	2
Decreased appetite	24	15	0	0
Pyrexia	40	34	0	0
Constipation	33	26	0	0
Nausea	6	4	0	0
Hand–foot skin reaction	6	4	0	0
Hypothyroidism	23	16	1	0
Hemorrhage from gastric varices	1	1	0	0

## Discussion

HCC is an aggressive malignancy characterized by a high propensity for recurrence and prompt disease progression (Freddie et al., 2024). As the current standard of care for unresectable HCC (uHCC), TACE has a noticeable function in managing this hypervascular tumor. HCC typically exhibits remarkable arterial phase enhancement and rapid contrast washout during the portal phase on imaging, reflecting its rich blood supply (Atsushi et al., 2017). TACE exerts its therapeutic effects by inducing ischemic necrosis through selective occlusion of the tumor’s arterial blood flow. This method not only disrupts the tumor’s vascular supply, but also triggers the release of tumor-associated antigens following tumor cell destruction, contributing to the cytotoxic effect (Ren et al., 2021; Jenny et al., 2023). However, distant metastases typically remain unaffected by localized interventions, necessitating the exploration of combined locoregional and systemic therapeutic strategies to improve outcomes in HCC management.

ICIs, targeting the PD-1/PD-L1 signaling pathway, represent an evolving Frontier in cancer therapy (Liu et al., 2024). ICIs facilitate an enhanced infiltration of CD4^+^ and CD8^+^ T cells in the tumor microenvironment, contributing to an amplified immune response (Kikuchi et al., 2022). CD8^+^ can completely wipe out malignancy cells by means of cell lysis and apoptosis (Jin et al., 2017). The antitumor function of CD8 + T cell recovered from PD-1 blockade (Se et al., 2016; Yu et al., 2024). The TACE treatment may enhance the number and function of CD8^+^ cells in HCC patients (Jingjing et al., 2021; Alessandro et al., 2006).

Despite the potential of immunotherapy, its efficacy is often compromised by the formation of an immunosuppressive microenvironment dominated by regulatory T (Treg) cells, which blunt anti-tumor immunity. TACE may potentiate the immune response by depleting Treg cells and modulating their immunosuppressive activities, thereby synergizing with immune checkpoint blockade (Ren et al., 2021). The targeted depletion of Tregs along with modulation of their functional activity through TACE can potentiate a more robust and efficacious anti-tumor immune response (Yuan et al., 2024). The combination of TACE with PD-1 inhibitors has exhibited to be not only safe, but also capable of significantly delaying tumor progression and reducing disease stage in carefully selected patients (Huang et al., 2024). Moreover, targeted therapies that inhibit FGF receptor 4 (FGFR4) can enhance the effectiveness of anti-PD-1 therapy by reducing PD-L1 expression and impeding Treg differentiation, further augmenting the anti-tumor immune response (Yi et al., 2021).

HCC could be treated by adjusting the proportion of the Kupffer macrophage, the treatment of TACE could decrease the expression of CYP3A4 with HepG2 HCC cells (Alican et al., 2023). There were Kupffer cells (KCs) expressed high levels of B7-H1 and CD8^+^ T cells with high expression of PD-1 in the HCC stroma. Inhibitory B7-H1/PD-1 interaction could improve the effector function of T cells (Ke et al., 2009).

In this investigation, the T+T+I treatment regimen demonstrated a substantial survival benefit relative to the T+T regimen, both before and after PSM. Post-PSM analysis unveiled that the T+T+I group achieved a mPFS of 17 months and a mOS of 28 months, relative to 12 months (mPFS) and 19 months (mOS) in the T+T group (P = 0.023 for mPFS; P = 0.034 for mOS). Furthermore, multivariate analysis identified the T+T+I treatment as an independent prognostic factor for prolonged PFS and OS, emphasizing its superior efficacy. Consequently, the findings revealed that the T+T+I regimen provided a significant therapeutic advantage over T+T as a first-line treatment option for unresectable HCC cases.

In the CHANCE2211 study, a cohort of 26 cases with intermediate-to-advanced HCC who underwent TACE plus camrelizumab and apatinib demonstrated markedly prolonged median PFS (13.5 vs. 7.7 months) and median OS (24.1 vs. 15.7 months) relative to those treated with monotherapy (Jin et al., 2023). Dawood et al. (2024) corroborated these findings, reporting that the tri-modal approach integrating ICIs, TKIs, and TACE conferred superior tumor response rates and enhanced survival outcomes in HCC cases, paralleling the outcomes of this investigation.

Nonetheless, this investigation is not without limitations. First, although PSM was employed to mitigate potential confounding variables, the inherently retrospective design might introduce the possibility of residual selection bias, which could not be entirely eliminated. Therefore, in the next study, all available covariates should be performed more sample heterogeneity analysis via performing PCA analysis. The lasso regression method should be used to reduce the multicollinearity between covariables. In the future, external databases should be used to support research. In addition, the subsequent treatment methods may affect the patients’ prognosis. This study only utilized PD-1 inhibitors and did not employ other immune checkpoint inhibitors. The enrolled patients lacked PD-1 expression. Thus, the prospective clinical studies will further explore the efficacy of targeted drugs combined with other immunosuppressants, such as PD-1, PD-L1 and CTLA-4. The expression of PD-1 in the patients who participated in the study was followed by PD-1 staining of the pathological sections of the patients and IHC scoring. Finally, differences in various TKIs and ICIs used may potentially confound the results. According to the findings so far, the future prospective studies may further explore a target combined with a PD-1 combined with TACE to corroborate the findings. Thus, precisely designed prospective trials are imperative to robustly validate these preliminary conclusions.

AEs reported in this investigation were largely manageable, with no instances of fatal outcomes, and the incidence rates of AEs aligned with those previously documented (Deng et al., 2024.). Notably, the data suggests that the T+T+I regimen did not exacerbate the risk of TRAEs relative to the T+T approach.

## Conclusion

In conclusion, incorporating TACE, TKI, and ICI remarkably enhanced both PFS and OS relative to TACE with TKI alone, positioning it as a more efficacious first-line therapeutic strategy for unresectable HCC, while maintaining an acceptable safety profile in clinical settings.

## Data Availability

The original contributions presented in the study are included in the article/supplementary material, further inquiries can be directed to the corresponding author.
